# Presence of Bromotyrosine Alkaloids in Marine Sponges Is Independent of Metabolomic and Microbiome Architectures

**DOI:** 10.1128/mSystems.01387-20

**Published:** 2021-03-16

**Authors:** Ipsita Mohanty, Subhasish Tapadar, Samuel G. Moore, Jason S. Biggs, Christopher J. Freeman, David A. Gaul, Neha Garg, Vinayak Agarwal

**Affiliations:** a School of Chemistry and Biochemistry, Georgia Institute of Technology, Atlanta, Georgia, USA; b University of Guam Marine Laboratory, UOG Station, Mangilao, Guam; c Department of Biology, College of Charleston, Charleston, South Carolina, USA; d Smithsonian Marine Station, Ft. Pierce, Florida, USA; e School of Biological Sciences, Georgia Institute of Technology, Atlanta, Georgia, USA; University of California, Berkeley

**Keywords:** marine sponge, metabolomics, mass spectrometry, microbiome, natural products

## Abstract

Marine sponge holobionts are prolific sources of natural products. One of the most geographically widespread classes of sponge-derived natural products is the bromotyrosine alkaloids. A distinguishing feature of bromotyrosine alkaloids is that they are present in phylogenetically disparate sponges. In this study, using sponge specimens collected from Guam, the Solomon Islands, the Florida Keys, and Puerto Rico, we queried whether the presence of bromotyrosine alkaloids potentiates metabolomic and microbiome conservation among geographically distant and phylogenetically different marine sponges. A multi-omic characterization of sponge holobionts revealed vastly different metabolomic and microbiome architectures among different bromotyrosine alkaloid-harboring sponges. However, we find statistically significant correlations between the microbiomes and metabolomes, signifying that the microbiome plays an important role in shaping the overall metabolome, even in low-microbial-abundance sponges. Molecules mined from the polar metabolomes of these sponges revealed conservation of biosynthetic logic between bromotyrosine alkaloids and brominated pyrrole-imidazole alkaloids, another class of marine sponge-derived natural products. In light of prior findings postulating the sponge host itself to be the biosynthetic source of bromotyrosine alkaloids, our data now set the stage for investigating the causal relationships that dictate the microbiome-metabolome interconnectedness for marine sponges in which the microbiome may not contribute to natural product biogenesis.

**IMPORTANCE** Our work demonstrates that phylogenetically and geographically distant sponges with very different microbiomes can harbor natural product chemical classes that are united in their core chemical structures and biosynthetic logic. Furthermore, we show that independent of geographical dispersion, natural product chemistry, and microbial abundance, overall sponge metabolomes tightly correlate with their microbiomes.

## INTRODUCTION

Marine sponges are holobionts in which a eukaryotic host is associated with a rich and diverse microbiome. These filter-feeding sessile benthic invertebrates are validated sources of bioactive small organic molecules, colloquially referred to as natural products. Several thousand sponge-derived natural products have been described. Interest in sponge-sourced natural products is sustained due to their pharmaceutical potential and their roles in shaping benthic marine community structure (1, 2). Building upon spectroscopic and crystallographic descriptions of sponge-derived natural products, mass spectrometry (MS)-based metabolome mining strategies are beginning to reveal additional chemical diversity in sponge holobionts (3, 4).

**FIG 1:**
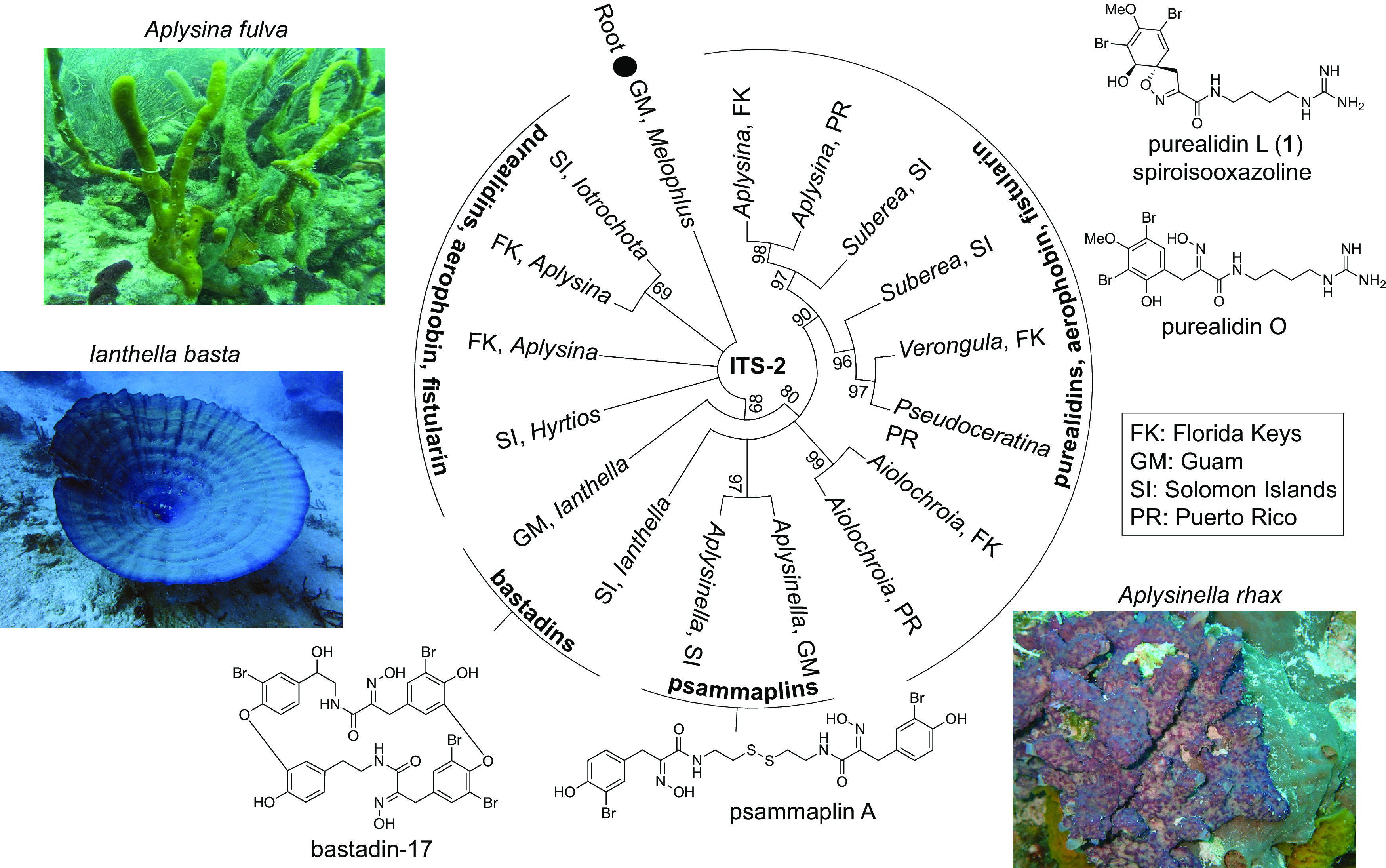
Sponge phylogeny and bromotyrosine alkaloid natural product subclasses. The distinctive morphology of some sponge specimens allows for species assignment. Illustrated here are Aplysina fulva (collected in the Florida Keys), Ianthella basta (Guam), and Aplysinella rhax (Guam). Representative structures of bromotyrosine alkaloids detected in each specimen are shown.

One of the most abundant, structurally diverse, and geographically ubiquitous classes of marine sponge-derived natural products is the bromotyrosine alkaloids; over 800 congeners are now known (5, 6). Retrosynthetically, this chemical diversity can be recapitulated by a single unifying principle: the condensation of a hydroxyimino bromotyrosine with an alkaloid that is derived from the decarboxylation of (non)proteinogenic amino acids. Based on this rationalization, bromotyrosine alkaloids can be divided into three subclasses, as illustrated in Fig. 1. The first and geographically most dispersed group consists of bromotyrosine alkaloids in which tyrosine-derived ketoxime carboxylic acids are condensed with aminoacyl alkaloids. In such molecules, the phenyl ring is routinely hydroxylated, and they are typified by the purealidins, aerophobins, and fistularins, which often coexist with their isomeric spirocyclohexadienylisooxazoline congeners (henceforth referred to as spiroisooxazoline bromotyrosine alkaloids for simplicity). Methylation or aminopropylation of the dibrominated phenoxyl is also commonly observed. The second subclass of molecules is the bastadins, wherein bromotyrosine ketoximes are condensed with a brominated tyramine followed by biaryl couplings to generate symmetric and asymmetric dimers (7). Unlike the first subclass, which is detected in various sponge genera, bastadins are limited to *Ianthella*, a sponge genus localized to the Indo-Pacific Ocean (Fig. 1). The third subclass of molecules is the psammaplins, wherein monobrominated tyrosine ketoximes are condensed with cysteamine followed by dimerization around a disulfide linkage. For bastadins and psammaplins, sulfation of the bromotyrosine phenoxyl has been observed.

The chemical transformation of aerophobins and psammaplins in response to sponge tissue damage posits a role for bromotyrosine alkaloids as chemical defenses in their native environments (8–11). Indeed, hydroxyimino aromatic amino acids serve as defense compounds in terrestrial plants (12). Sponges bearing bromotyrosine alkaloids emit hydrogen cyanide when damaged, which is also a hallmark of plant hydroxyimino tyrosine-derived cyanogenic glucosides such as dhurrin (13, 14). The pharmacology of bastadins and psammaplins is well developed, with the former acting as ryanodine receptor agonists and the latter acting as histone deacetylase inhibitors in a manner reminiscent of other metal-chelating marine prodrug thiols such as romidepsin and largazole (15–18). Despite their ubiquity, ecological roles, and pharmacological potential, biosynthetic routes to bromotyrosine alkaloids have not been described.

Driven by contemporary metagenomic sequencing, assembly, and genome mining approaches, the prevailing view of natural product biosynthesis in sponge holobionts is that the bacterial symbionts associated with the sponge eukaryotic host are the engines for the production of natural products. In all cases where the biogenetic routes for marine holobiont-derived natural products have been described, sponge or otherwise, the microbiome structure remains correlatively conserved with the natural product chemistry (4, 19). The presence of bromotyrosine alkaloids is spread across numerous geographically disperse sponges of different morphologies and phylogenies. In light of this spread, it is not currently understood if there are common microbiome features that underlie the presence of bromotyrosine alkaloids in sponge holobionts. Furthermore, metabolomic conservation among phylogenetically and geographically distant sponges that harbor bromotyrosine alkaloids has not been mapped. Here, we provide a paired multi-omic characterization of bromotyrosine alkaloid-bearing sponges to reveal vastly different microbiome architectures that are tightly correlated with disparate metabolomes of sponge holobionts. In addition to an inventory of natural products, mining the polar metabolomes of these marine sponges yields biosynthetic insights for the construction of bromotyrosine alkaloids together with the curious conservation of bioactive betaines that have otherwise been shown to mediate interorganismal interactions in the terrestrial environment.

## RESULTS AND DISCUSSION

### Diverse sponge genera host bromotyrosine alkaloids.

A library of sponge specimens from Guam, the Solomon Islands, the Florida Keys, and Puerto Rico (abbreviated GM, SI, FK, and PR, respectively, in Fig. 1) was assembled (see Table S1 in the supplemental material). Sponge phylogenies were determined by Sanger sequencing of PCR amplicons for internal transcribed spacer 2 (ITS-2) and the 5′ terminus of the 28S rRNA gene. Species-level designations of marine sponges based entirely upon sequencing of PCR amplicons are not robust (20). Hence, phylogenetic descriptions of specimens determined by this study were restricted to genera. Multiple specimens of the same genus from one geographical region, such as *Aplysina* from the Florida Keys, represent morphologically distinct species. Separate phylogenetic trees were constructed with the ITS-2 and 28S amplicon sequences using the corresponding sequences for the sponge Melophlus sarasinorum collected from Guam as the root; M. sarasinorum does not possess brominated alkaloids (20). Bootstrap values thus obtained demonstrated that the phylogenetic grouping using ITS-2 sequences was better supported than that based on 28S amplicon sequences (Fig. 1; Fig. S1).

10.1128/mSystems.01387-20.1 FIG S1 Phylogenetic tree constructed using the 28S ribosomal PCR amplicons. Download FIG S1, EPS file, 1.6 MB. Copyright © 2021 Mohanty et al. 2021 Mohanty et al. https://creativecommons.org/licenses/by/4.0/ This content is distributed under the terms of the Creative Commons Attribution 4.0 International license.

10.1128/mSystems.01387-20.10 TABLE S1 Phylogeny of sponge specimens used in this study as determined by consensus ITS-2 and 28S sequences. Download Table S1, DOCX file, 0.01 MB. Copyright © 2021 Mohanty et al. 2021 Mohanty et al. https://creativecommons.org/licenses/by/4.0/ This content is distributed under the terms of the Creative Commons Attribution 4.0 International license.

In the phylogenetic tree, we observed that sponge genera grouped according to the bromotyrosine alkaloid subclass (Fig. 1). Organic extracts of sponge biomass were analyzed by liquid chromatography-mass spectrometry (LC/MS). Chromatographic conditions employed preferentially retained nonpolar molecules; henceforth, these data are referred to as the nonpolar metabolomes. Typified by the characteristic isotopic signature of brominated molecules and manual annotation of MS^2^ fragment ions, the structures of previously described bromotyrosine alkaloids were dereplicated from the natural products database MarinLit (http://pubs.rsc.org/marinlit/). In *Ianthella* and *Aplysinella* specimens collected from Guam and the Solomon Islands, bastadins and psammaplins were detected, respectively (Fig. S2). All other specimens of diverse genera such as *Aplysina*, *Verongula*, *Suberea*, *Pseudoceratina*, and *Aiolochroia* were found to contain the spiroisooxazoline alkaloids. To differentiate between the spiroisooxazolines and the isomeric phenyl 3′-hydroxylated structures (for example, isomeric purealidin L [compound 1] and purealidin O illustrated in Fig. 1), compound 1 was isolated from an *Aplysina* specimen collected in the Florida Keys and structurally confirmed by comparison of ^1^H and ^13^C NMR (nuclear magnetic resonance) shifts to those reported in the literature (Fig. S3) (21).

10.1128/mSystems.01387-20.2 FIG S2 Structural annotation of MS^2^ spectra for the three classes of bromotyrosine alkaloids. Download FIG S2, EPS file, 2.2 MB. Copyright © 2021 Mohanty et al. 2021 Mohanty et al. https://creativecommons.org/licenses/by/4.0/ This content is distributed under the terms of the Creative Commons Attribution 4.0 International license.

10.1128/mSystems.01387-20.3 FIG S3 ^1^H NMR (DMSO-*d*_6_) (top) and ^13^C NMR (in CD_3_OD) (bottom) spectra for compound 1. Download FIG S3, PDF file, 0.6 MB. Copyright © 2021 Mohanty et al. 2021 Mohanty et al. https://creativecommons.org/licenses/by/4.0/ This content is distributed under the terms of the Creative Commons Attribution 4.0 International license.

Bromotyrosine alkaloid-harboring sponges collected as a part of this study fell into three different orders within the class Demospongiae (Table S1). Specimens were further divided among six different families, four of which are within the order Verongiida, and nine genera collected from four different locations. Among natural products, the geographical and phylogenetic diversity of sponges harboring bromotyrosine alkaloids is perhaps equaled only by sponge-derived meroterpenes (22), triterpenoids, and polyacetylenic alcohols and acids (23). No natural products that have been demonstrated to be biosynthesized by sponge symbiotic bacteria are as widely distributed among disperse sponge clades (4, 19). In *Aplysina* sponges, bromotyrosines are found localized within the spherulous cells in the sponge mesohyl, supporting the postulate that the sponge host itself, and not the bacterial symbionts, may be the biogenetic source of bromotyrosine alkaloids (24–26).

### Nonconvergent microbiome architectures.

Next, we determined the microbiome architectures for all bromotyrosine alkaloid-bearing sponge specimens. For marine sponges, microbial diversity correlates with microbial abundance; diverse microbiomes are indicative of high microbial abundance (HMA), while lesser diversity signifies low microbial abundance (LMA) (27). Bacterial 16S amplicon sequencing for all samples in this study was conducted in parallel. We also included three sponges that do not possess bromotyrosine alkaloids as controls in this analysis. Control sponges include *Smenospongia* (collected in the Florida Keys) (Table S1), *Stylissa* (Guam), and *Agelas* (Solomon Islands). The metabolomes of these control sponges have been previously described: *Stylissa* and *Agelas* contain the brominated pyrrole-imidazole alkaloids, while *Smenospongia* harbors the brominated indoles and cyanobacterial symbiont-derived chlorinated hybrid peptides/polyketides (28, 29). Among these controls, *Smenospongia* and *Agelas* are HMA sponges, while *Stylissa* is an LMA sponge (27, 29).

**FIG 2:**
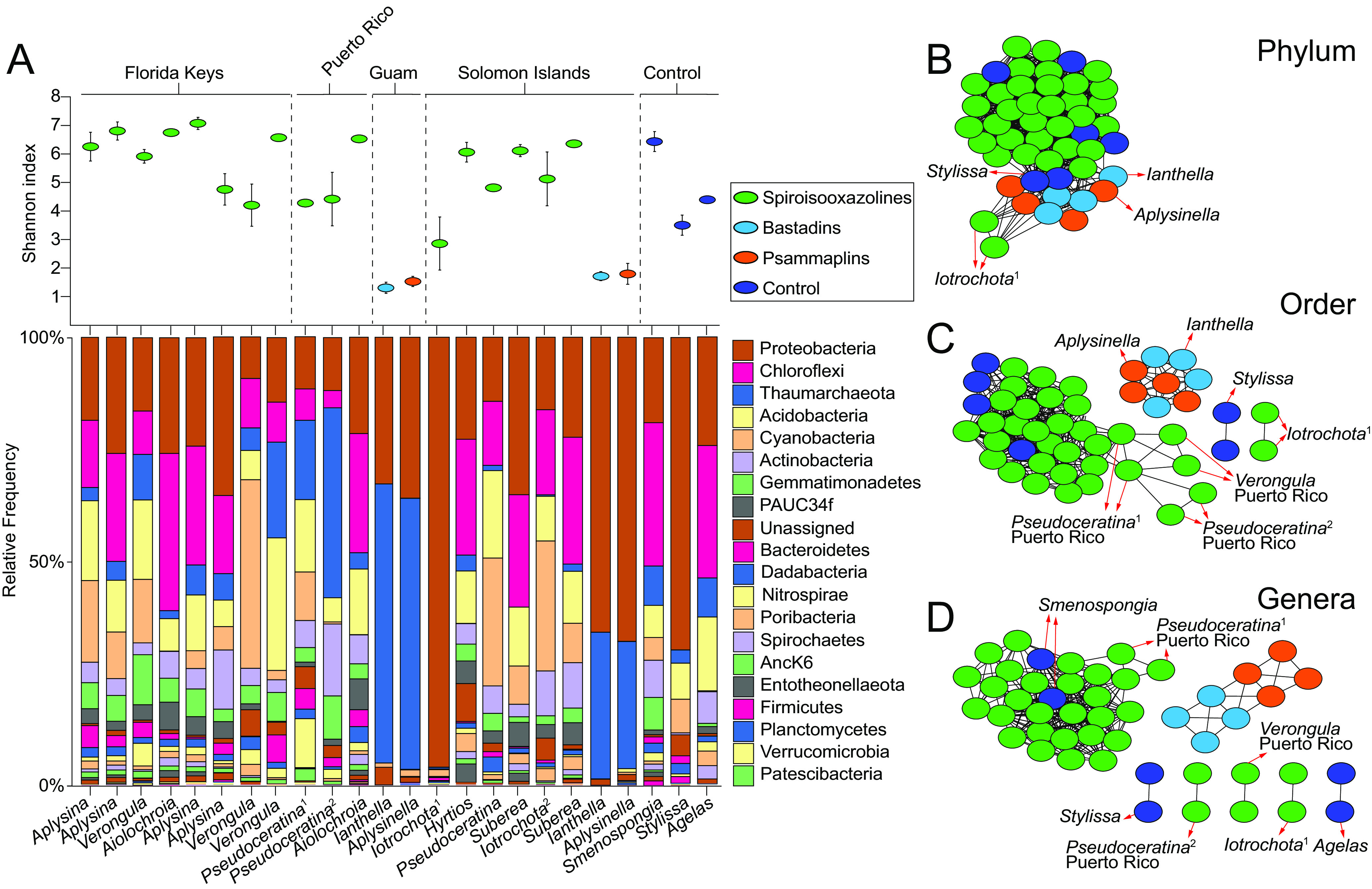
(A) Shannon indices (denoting α-diversity) and distribution of bacterial phyla in microbiomes of bromotyrosine alkaloid-harboring sponge specimens used in this study. Superscript numbers denote specimens of same genus but different species collected from the same geographical site. (B to D) Bray-Curtis dissimilarity networks at the phylum (B), order (C), and genus (D) levels for the sponge microbiomes. Sponge microbiomes were sequenced in duplicate. The connectivity cutoff was set at 0.6; nodes with dissimilarity indices of less than 0.6 are connected with the edge length indicative of the dissimilarity index magnitude.

Plotting the Shannon indices demonstrated that *Ianthella* and *Aplysinella* specimens collected in Guam and the Solomon Islands, which harbor the bastadins and psammaplins, respectively, were distinctively LMA sponges (Fig. 2A). With the exception of an *Iotrochota* specimen collected in the Solomon Islands, all sponges that possess the spiroisooxazoline alkaloids were HMA sponges. The presence of bacteria of the phylum *Chloroflexi* was characteristic of HMA sponges, as has been described previously (30).

Next, we generated two-dimensional Bray-Curtis dissimilarity matrices for sponge microbiomes at the phylum, order, and genus levels. These matrices are represented as networks in which nodes with dissimilarity indices of <0.6 are interconnected with edge length, indicative of the magnitude of the dissimilarity index (Fig. 2B to D). Each microbiome was sequenced in duplicate; hence, each sponge specimen is represented by two nodes in these networks. At the phylum level, all nodes were interconnected (Fig. 2B). However, nodes corresponding to the LMA sponges *Iotrochota*, *Ianthella*, and *Aplysinella* were starting to separate along with the LMA control sponge *Stylissa*.

At the order level, *Ianthella* and *Aplysinella* formed a separate subnetwork, while the LMA *Iotrochota* nodes disassociated completely (Fig. 2C). Illustrative to note is that the nodes for the control LMA sponge *Stylissa* also disassociated. However, nodes for the HMA control sponges *Agelas* and *Smenospongia* remained interconnected with other bromotyrosine alkaloid-harboring sponges. Disassociation from the network correlated with α-diversity while being independent of natural product chemistry; the four sponge genera with the lowest Shannon indices separated from the parent network.

At the genus level, further disassociation of nodes for sponges with the next-higher Shannon indices, such as the control sponge *Agelas*, was observed (Fig. 2D). The HMA control sponge *Smenospongia* remained interconnected with other bromotyrosine alkaloid-bearing sponge genera such as *Aplysina*, *Verongula*, *Aiolochroia*, and *Suberea*. For the spiroisooxazolines, there is only limited conservation of microbiomes. While a majority of sponges harboring this subclass of bromotyrosine alkaloids are HMA sponges, some, such as *Iotrochota*, *Verongula*, and *Pseudoceratina*, possess distinctively different microbiome architectures (Fig. 2D).

### Detection of rationalized biosynthetic intermediates.

**FIG 3:**
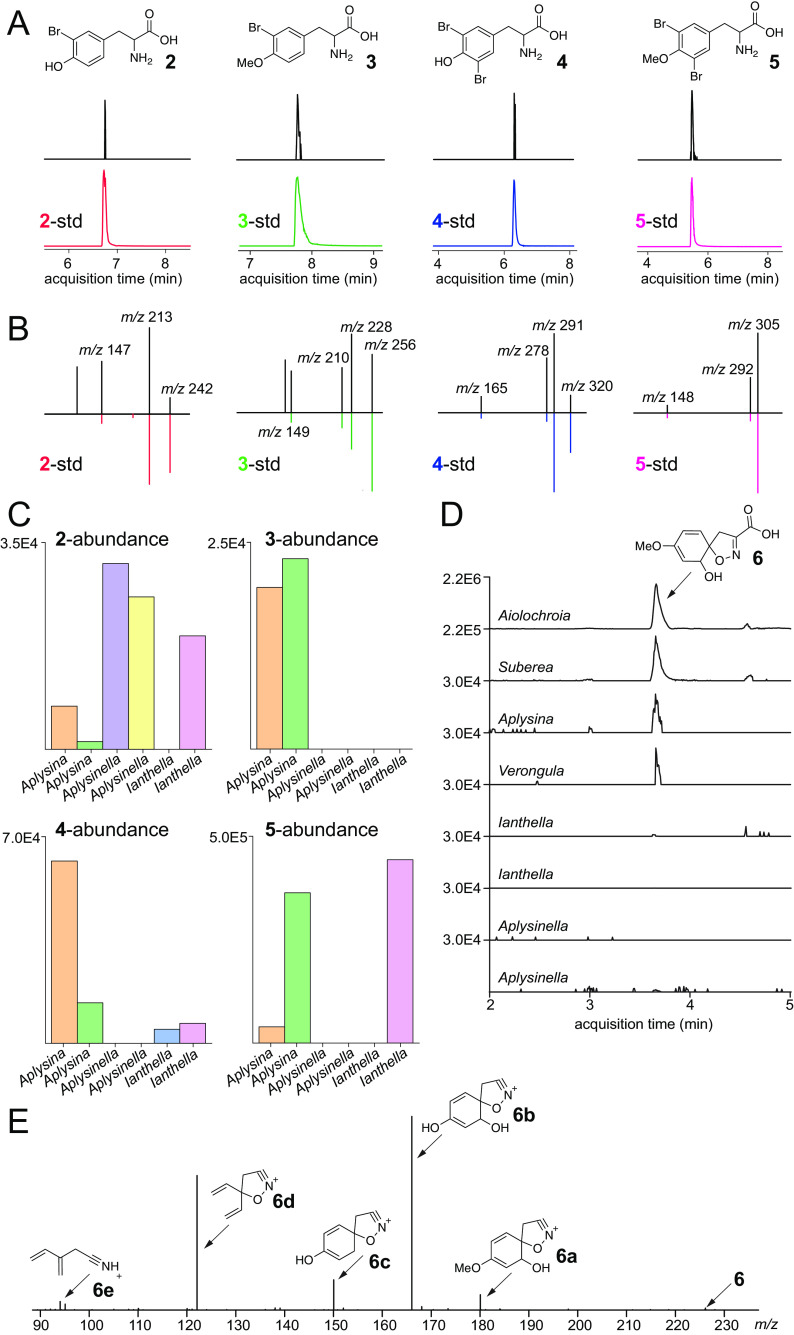
(A to C) Extracted ion chromatogram (EIC) retention time matches (A), MS^2^ spectral matches (B), and relative abundances (C) for bromotyrosines 2 to 5 monitored for two *Aplysina*, *Aplysinella*, and *Ianthella* specimens. Panel A represents EICs generated within a ±0.01-Da mass tolerance for the most abundant [M + H]^1+^ isotopic ion. The metabolite abundance in panel C was calculated by the integration of the EIC peak for the [M + H]^1+^ ion. (D and E) EIC for the [M + H]^1+^ ion corresponding to compound 6 (D) and MS^2^ spectra for compound 6 (E). Structural annotations for MS^2^ fragments are all within a 2-ppm error.

The biosynthesis of the three different subclasses of bromotyrosine alkaloids can be rationalized to involve common intermediates. Among the psammaplins, it is instructive to observe that the tyrosine unit is always monobrominated; no dibrominated psammaplin congeners have been detected. Unlike the spiroisooxazolines in which the dibrominated phenoxyl is always methylated, methoxylated psammaplins are likewise not detected. In contrast to the psammaplins, both mono- and dibrominated tyrosine building blocks can be rationalized from the inventory of bastadin structures, while phenyl methoxylation is observed for hemibastadins (6, 7). To query whether these structural observations are predictive of the corresponding biosynthetic intermediates, bromotyrosines 2 to 5 were synthesized and used as standards to query the metabolomes of two specimens each of *Aplysina*, *Aplysinella*, and *Ianthella* sponges that bear the spiroisooxazolines, psammaplins, and bastadins, respectively (Fig. 3; Fig. S4 to S7). Sponge extracts were analyzed by LC/MS using modified chromatographic procedures designed to retain polar metabolites; these data are henceforth referred to as the polar metabolomes. Metabolite identification in these polar metabolomes relied on the matching of LC retention times and MS^2^ fragmentation spectra to synthetic standards.

10.1128/mSystems.01387-20.4 FIG S4 ^1^H NMR spectrum for compound 2 in CD_3_OD. Download FIG S4, EPS file, 1.7 MB. Copyright © 2021 Mohanty et al. 2021 Mohanty et al. https://creativecommons.org/licenses/by/4.0/ This content is distributed under the terms of the Creative Commons Attribution 4.0 International license.

10.1128/mSystems.01387-20.5 FIG S5 ^1^H NMR spectrum for compound 3 in CD_3_OD. Download FIG S5, EPS file, 1.7 MB. Copyright © 2021 Mohanty et al. 2021 Mohanty et al. https://creativecommons.org/licenses/by/4.0/ This content is distributed under the terms of the Creative Commons Attribution 4.0 International license.

10.1128/mSystems.01387-20.6 FIG S6 ^1^H NMR spectrum for compound 4 in CD_3_OD. Download FIG S6, EPS file, 1.7 MB. Copyright © 2021 Mohanty et al. 2021 Mohanty et al. https://creativecommons.org/licenses/by/4.0/ This content is distributed under the terms of the Creative Commons Attribution 4.0 International license.

10.1128/mSystems.01387-20.7 FIG S7 Synthetic scheme (top) and ^1^H NMR spectrum (bottom) for compound 5 in CD_3_OD. Download FIG S7, EPS file, 1.8 MB. Copyright © 2021 Mohanty et al. 2021 Mohanty et al. https://creativecommons.org/licenses/by/4.0/ This content is distributed under the terms of the Creative Commons Attribution 4.0 International license.

Bromotyrosine building blocks could be detected in all three sponge genera (Fig. 3). Consistent with the observed structural features of psammaplins, dibrominated compounds 4 and 5 or methoxylated compound 3 was not detected in either of the two *Aplysinella* specimens. *Aplysina* possessed the greatest diversity, wherein each of the four molecules 2 to 5 was detected. While spiroisooxazolines are always dibrominated, monobrominated compounds 2 and 3 can be rationalized as precursors for compounds 4 and 5. The sequence of bromination and methylation in the biosynthetic scheme can be interchanged. Halogenases participating in the biosynthesis of compounds 2 to 5 are likely obligate brominases; corresponding chlorinated congeners were not detected in any of the polar metabolomes. It is as yet unclear if bromination is affected by free tyrosine or by tyrosine that is thioesterified to carrier proteins, as is the case for the proposed biosynthetic scheme for bromoalterochromides, the only bromotyrosine-containing natural products for which the identification of the biosynthetic genes has been experimentally validated (31, 32). Among Verongiida sponges, bromotyrosines are also constituents of the collagenous spongin-chitin skeleton (33, 34), although the extraction procedures used in this study bias the detection of bromotyrosines from the sponge tissue rather than from the recalcitrant skeletal polymers.

Is tyrosine bromination an early- or a late-stage biosynthetic event? While the detection of compounds 2 to 5 indeed points toward brominated tyrosines being available for downstream biosynthetic enzymes, we also queried alternate hypotheses. Mining the polar metabolomes for MS^1^ [M + H]^1+^ ions corresponding to compound 6 led to its observation in the *Aiolochroia*, *Suberea*, *Aplysina*, and *Verongula* genera (Fig. 3D). While we could not synthesize a standard for compound 6, annotation of the MS^2^ spectra is supportive of the structural assignment (Fig. 3E). Specifically, fragmentation ions 6a and 6b support the presence of carboxylate and methyl moieties. In the MS^2^ spectra for amino acids, oxidative deamination and the neutral loss of the —CONH_2_ group from C_α_ are commonly observed (29, 35). However, both these modifications are absent in the fragmentation spectra for compound 6, pointing toward modification of the primary amine that we hypothesize to be involved in the formation of the isooxazoline ring with tyrosine C_α_, C_β_, and C_γ_ atoms, which is also supported by the structural assignment of fragmentation ions 6c to 6e. MS^1^ ions corresponding to compound 6 are not detected in either of the two specimens of *Ianthella* and *Aplysinella* (Fig. 3D); bastadins and psammaplins do not possess the spirocyclohexadienylisooxazoline building block.

**FIG 4:**
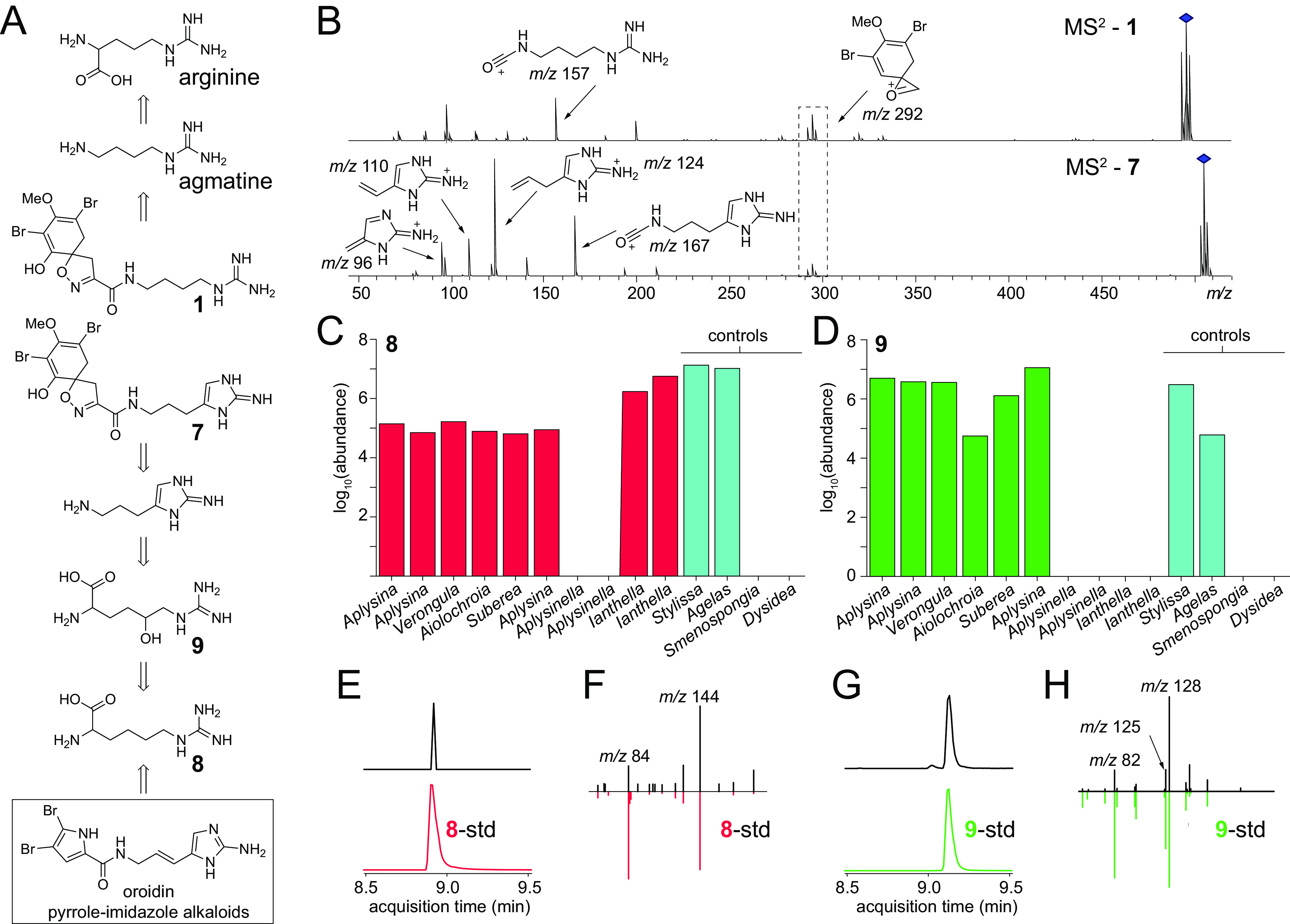
(A) Rationalized retrobiosynthetic scheme for the elaboration of the 2-aminoimidazole propylamine building block that is incorporated into bromotyrosine alkaloid 7 and in pyrrole-imidazole alkaloids such as oroidin. (B) Comparison of MS^2^ fragmentation spectra for compounds 1 and 7 demonstrating divergence in the aminoacyl alkaloid that is ligated to the conserved bromotyrosine building block. (C and D) Relative abundances of compound 8 (C) and compound 9 (D) in sponge extracts as determined by the integration of the EIC peak area generated from the LC/MS data. (E and F) EIC retention times (E) and MS^2^ spectrum matches (F) for compound 8 detected in sponge extracts against an authentic standard. (G and H) Similar to panels E and F, with EIC retention times (G) and MS^2^ spectrum matches (H) for compound 9 detected in sponge extracts against a racemic synthetic standard.

The rationalized inventory of biosynthetic precursors for bastadins and psammaplins is sparse, restricted to tyrosine and tyramine for bastadins and tyrosine and cysteamine for psammaplins. The chemical diversity of these two subclasses of bromotyrosine alkaloids is generated by further modification of the bromotyrosine-tyramine (bastadins) and bromotyrosine-cysteamine (psammaplins) conjugates. These modifications include phenoxyl sulfation and methylation, aminoacyl β-hydroxylation and dehydrogenation, and oxidative biaryl and disulfide couplings (6). In contrast, the inventory of rationalized biosynthetic precursors for spiroisooxazolines is larger. Here, diverse decarboxylated aminoacyl alkaloids are ligated to the dibrominated tyrosine-derived building block. For sponges used in this study, aminoacyl alkaloids conceivably derived from the decarboxylation of histidine, lysine, and ornithine can be discerned by manual annotation of the MS^2^ spectra (Fig. S8). For compound 1, detected in high abundances in *Aplysina*, *Verongula*, *Aiolochroia*, and *Suberea* specimens collected in the Florida Keys, Puerto Rico, and the Solomon Islands, the aminoacyl building block agmatine can be rationalized to be derived via the decarboxylation of arginine (Fig. 4A). In these sponge metabolomes, together with compound 1, aerophobin-2 (compound 7) (Fig. 4A) is also detected. Comparison of MS^2^ spectra for compounds 1 and 7 demonstrates that the dibrominated tyrosine building block is conserved between the two structures (Fig. 4B). However, unlike compound 1, the aminoimidazole propylamine moiety for compound 7 cannot be rationalized starting from proteinogenic and commonly encountered nonproteinogenic amino acids.

10.1128/mSystems.01387-20.8 FIG S8 Structural annotation of MS^2^ spectra for the bromotyrosine alkaloids detected in sponge genus *Aplysina*. Download FIG S8, EPS file, 2.1 MB. Copyright © 2021 Mohanty et al. 2021 Mohanty et al. https://creativecommons.org/licenses/by/4.0/ This content is distributed under the terms of the Creative Commons Attribution 4.0 International license.

We recently reported that the structurally similar aminoimidazole propylamine precursor for the sponge-derived natural product oroidin is likely derived from the nonproteinogenic amino acid homoarginine (compound 8) (Fig. 4A) (29). Oroidin and its desbromo congeners hymenidin and clathrodin undergo intramolecular cyclization and homo- and hetero-cycloadditive dimerization to furnish the pyrrole-imidazole alkaloids (36). We had hypothesized that compound 8 undergoes C`συβ>δ`/συβ> hydroxylation to furnish compound 9. Oxidation of the secondary alcohol can lead to spontaneous dehydration to install the aminoimidazole heterocycle. This oxidative dehydration can occur before or after decarboxylation at C_α_ to afford the aminoimidazole propylamine moiety that is observed in compound 7 and, after further C_β_ dehydrogenation, in oroidin (Fig. 4A).

The above-mentioned hypothesis necessitates the presence of the nonproteinogenic amino acid 8 and its hydroxylated derivative compound 9 in sponge samples in which we detect the presence of compound 7. The presence of compounds 8 and 9 was queried for in polar metabolomes of various sponge specimens in which compound 7 was detected along with *Aplysinella* (containing psammaplins) and *Ianthella* (containing bastadins), in which compound 7 was not detected. Four other sponge specimens of the genera *Stylissa*, *Agelas*, *Smenospongia*, and *Dysidea* acted as controls in this workflow (Fig. 4C and D). We have demonstrated the presence of compounds 8 and 9 in *Stylissa* and *Agelas* previously and the absence of these metabolites in the *Dysidea* specimen used here (29). Due to the absence of pyrrole-imidazole and bromotyrosine alkaloids in *Smenospongia*, we anticipated that *Smenospongia* would not contain compounds 8 and 9.

In the polar metabolomes of control sponges, compounds 8 and 9 were detected in *Stylissa* and *Agelas* while being absent in *Dysidea* and *Smenospongia* (Fig. 4C and D). In all sponge tissues in which compound 7 was detected, we detected the presence of compounds 8 and 9 in high abundance; metabolite identification was based on comparison of retention times and MS^2^ spectra to a commercially available standard of compound 8 and a previously developed racemic standard of compound 9 (Fig. 4E to H) (29). Consistent with the absence of compound 7, *Aplysinella* specimens collected in Guam and the Solomon Islands did not contain either compound 8 or 9. Surprisingly, compound 8 was detected in high abundance in *Ianthella*; however, compound 9 was not detected. While the physiological role for compound 8 in *Ianthella* cannot be presently rationalized, the transformation of compound 8 to compound 9 appears to be a modification that is dedicated to the construction of the aminoimidazole propylamine building block. Our findings here extend the presence of compounds 8 and 9 to diverse sponge families and genera within the Verongiida order, in addition to the Scopalinida, Axinellida, and Agelasida orders from which we had described them previously (29).

It is instructive to observe the diversity of sponge microbiomes that harbor compound 8. Illustratively, the presence of compound 8 is conserved among LMA *Ianthella* and HMA *Aplysina* sponges that are at the extremities of the α-diversity plot and share little overlap among their microbiomes at the order and genus levels (Fig. 2). However, *Aplysinella* and *Ianthella*, both LMA sponges, are divergent in the presence of compound 8. Amino acid 8 can be generated by amidinotransfer upon lysine or by carbamylation of lysine to afford homocitrulline followed by amination. We could not detect homocitrulline in any of the polar metabolomes generated in this study. Carbamylation of lysine side chains is a nonenzymatic posttranslational modification associated with aging; it is unclear if this modification can occur on free lysine (37). On the other hand, biochemical precedence exists for amidinotransfer on free lysine to afford compound 8 (38, 39).

### Conservation of betaine chemistry.

**FIG 5:**
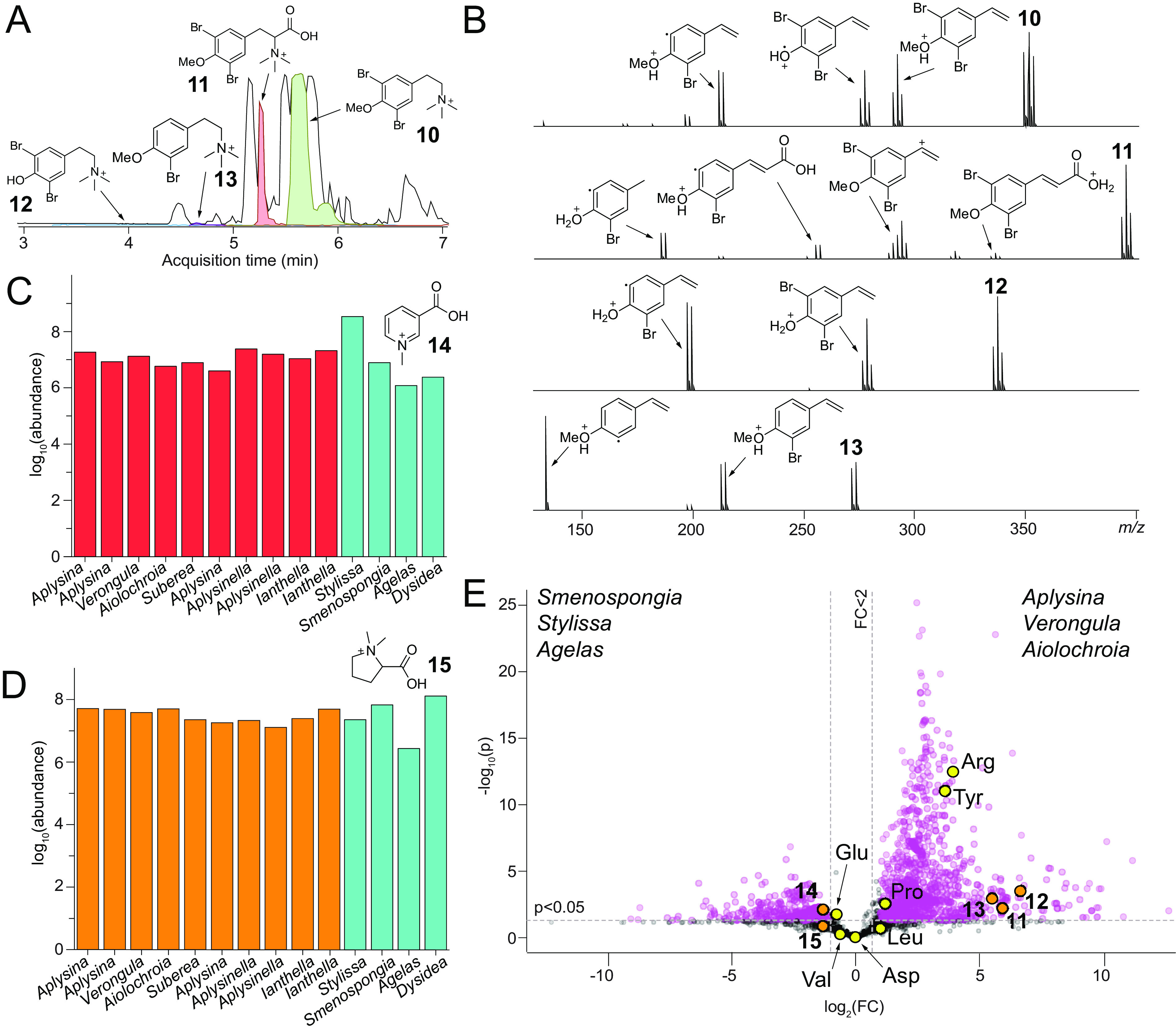
(A) Base peak chromatogram (in black) and superimposed EICs for compounds 10 to 13 in the nonpolar metabolome of an *Aplysina* sponge specimen. (B) Structural annotations of MS^2^ spectra for compounds 10 to 13. (C and D) Relative abundances of compound 14 (C) and compound 15 (D) in sponge polar metabolomes. (E) Comparison of sponge polar metabolomes, with nodes corresponding to betaines in orange and amino acids in yellow. FC, fold change.

In addition to biosynthetic precursors, another class of brominated alkaloids detected together with spiroisooxazolines in sponges is the tyramine- and tyrosine-derived betaines. In *Aplysina* specimens collected in the Florida Keys, previously reported betaines 10 and 11 were detected in high abundance (Fig. 5A) (40, 41). MS^2^ spectra for compounds 10 and 11 demonstrate the oxidative cleavage of trimethylamine and the radical scission of the carbon-bromine bonds being the dominant MS^2^ product ion-generating transformations (Fig. 5B). Guided by the structural annotation of the MS^2^ spectra, additional betaines such as compounds 12 and 13 could be detected in the sponge extracts. The abundance of compounds 12 and 13 was much lower than that of compounds 10 and 11; however, distinct retention times demonstrate that they are not in-source fragments generated in the mass spectrometer. Molecules 10 to 13 were detected in the nonpolar metabolomes, while compounds 11 to 13, but not compound 10, could be detected in the polar metabolomes.

In addition to betaines 10 to 13, in the polar metabolomes, we detected the presence of the betaines trigonelline (compound 14) and stachydrine (compound 15) (Fig. 5). As described above, compound identification relied on the comparison of retention times and MS^2^ spectra to synthetic standards (Fig. S9). Both compounds 14 and 15 were detected across all sponge specimens queried here, which include sponges bearing all three subclasses of bromotyrosine alkaloids and control sponges of the *Stylissa*, *Smenospongia*, *Agelas*, and *Dysidea* genera (Fig. 5C and D). Our data demonstrate that the presence of compounds 14 and 15 is broadly conserved in phylogenetically and geographically disperse HMA and LMA sponges. The presence of compounds 14 and 15 is not restricted to the marine environment: compound 14 has been demonstrated to mediate predator-prey interactions in aquatic systems, while compounds 14 and 15 mediate interactions between plants and their bacterial symbionts (42, 43). While the ecological roles of compounds 14 and 15 are appreciated in other diverse niches, their roles in sponge ecology are currently unknown.

10.1128/mSystems.01387-20.9 FIG S9 Retention times and MS^2^ spectral matches for compounds 14 and 15 between the *Aplysina* sponge extract and the respective synthetic standards. Download FIG S9, EPS file, 1.4 MB. Copyright © 2021 Mohanty et al. 2021 Mohanty et al. https://creativecommons.org/licenses/by/4.0/ This content is distributed under the terms of the Creative Commons Attribution 4.0 International license.

Next, we compared the polar metabolomes of *Aplysina*, *Verongula*, and *Aiolochroia* specimens collected in the Florida Keys to those of *Smenospongia*, *Stylissa*, and *Agelas* control sponges. These three control sponge genera are prolific producers of brominated metabolites, *Smenospongia* of brominated indoles and *Stylissa* and *Agelas* of brominated pyrrole-imidazole alkaloids. Here, we observed that betaines 11 to 13 were highly enriched in sponges containing the bromotyrosine alkaloids, while nonhalogenated betaines 14 and 15 were nearly equitably distributed among these diverse sponge genera (Fig. 5E). Note that compound 10 was not detected in polar metabolomes. Primary metabolites, such as amino acids (yellow nodes in Fig. 5E), acted as controls in this analysis. Although detected in high abundance in each polar metabolome, glutamate, valine, aspartate, leucine, and proline were not highly differentiating. However, we observed a relative enrichment of tyrosine and arginine in *Aplysina*, *Verongula*, and *Aiolochroia*, wherein they are proposed to act as precursors for the biosynthesis of compound 1 (Fig. 4A).

### Microbiome-metabolome interdependence.

The nonpolar metabolomes reported here are descriptive of the natural product chemistry harbored within the sponge holobionts, while the polar metabolomes capture biosynthetic intermediates and primary metabolites. Taken together, these data deliver a comprehensive metabolomic description for sponges that are united by the presence of bromotyrosine alkaloids. The biosyntheses of the three subclasses of bromotyrosine alkaloids share a central theme, that is, ligation of an aminoacyl alkaloid with a brominated tyrosine building block. Does this unified biosynthetic logic lead to overall metabolomic conservation among the sponge holobionts? We probed this question within the Verongiida order by comparing the nonpolar and polar metabolomes of sponges harboring the spiroisooxazoline (family Aplysinidae, various genera), bastadin (genus *Ianthella*), and psammaplin (genus *Aplysinella*) groups of bromotyrosine alkaloids by principal-coordinate analysis (PCoA). By restricting this analysis to only the Verongiida order, we intended to eliminate possible phylogenetic outliers.

**FIG 6:**
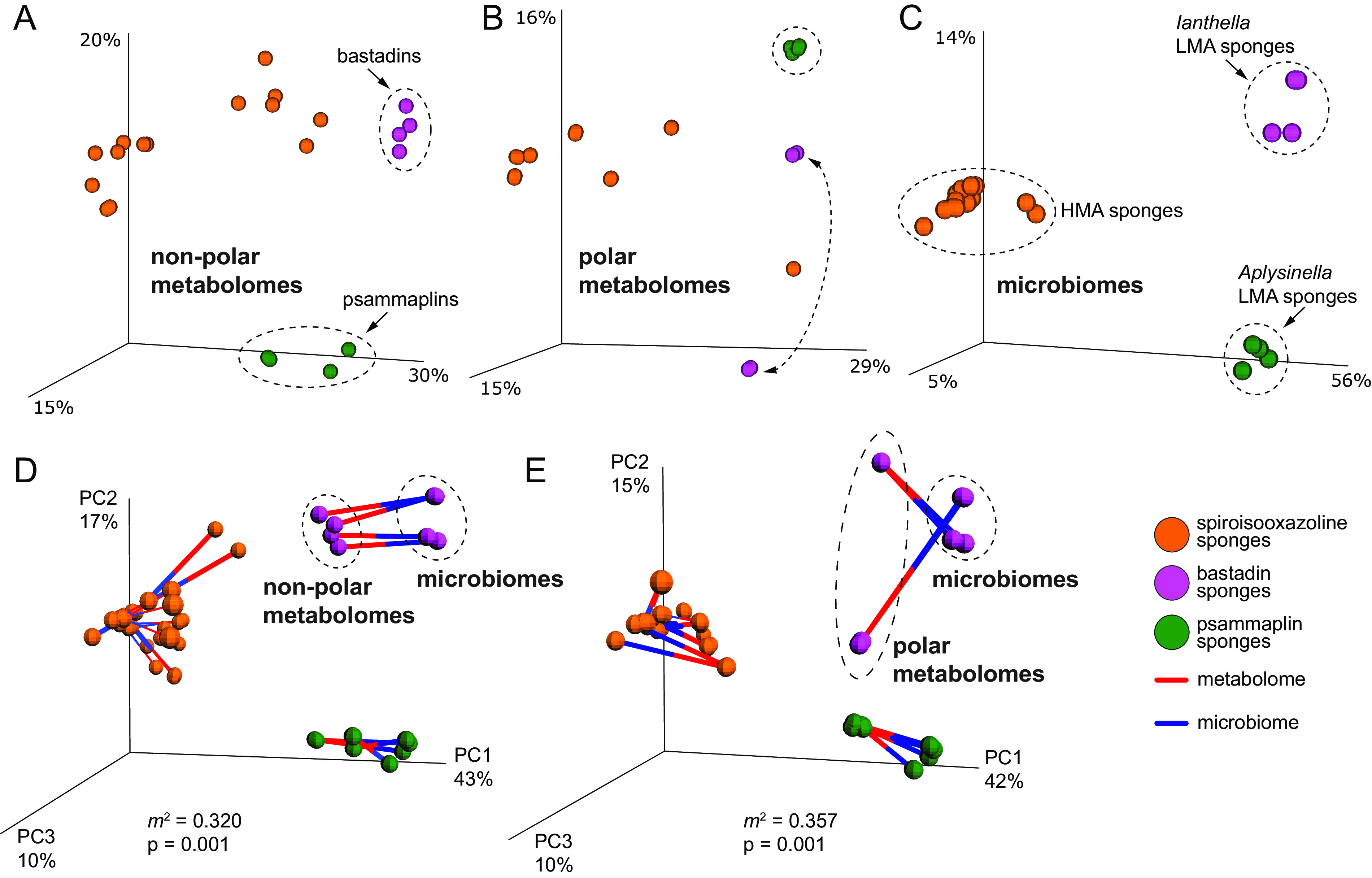
(A to C) Principal-coordinate score plots of nonpolar metabolomes (A), polar metabolomes (B), and microbiomes (C). The nodes are colored according to the subclass of bromotyrosine alkaloids. (D and E) Procrustes analyses of nonpolar metabolomes and microbiomes (D) and polar metabolomes and microbiomes (E). The blue edge-halves point toward microbiome nodes, while the red halves point toward the metabolomes.

In the PCoA plots, we observed significantly distant clustering of the nonpolar and polar metabolomes of the three sponge groups, with greater spread observed for the metabolomes of the spiroisooxazoline-harboring sponges (Fig. 6A and B). Notably, the polar metabolomes of the *Ianthella* samples collected in Guam and the Solomon Islands were distant, while the nonpolar metabolomes were indeed quite similar. Overall, distinct clusters for each sponge group were observed, with no overlap between groups. Next, the microbiomes were compared in a similar fashion. As for the metabolomes, the microbiomes neatly grouped into three separate and distant clusters (Fig. 6C).

To understand if the microbiome and metabolome divergences are correlated, we conducted a Procrustes analysis wherein we fit the PCoA matrices of the nonpolar and polar metabolomes to the microbiome matrix (Fig. 6D and E). For both the nonpolar and the polar metabolomes, we observed statistically significant correlations with the microbiome architecture, with the *m*^2^ Gower statistic in both instances being <0.5 (*P* < 0.001). In addition, clustering consistent with the bromotyrosine alkaloid subclass was still preserved in the Procrustes plots, with three distinct regions emerging in both cases. While it is conceivable that holobiont metabolomes will correlate with the microbiomes for HMA sponges such as those that harbor the spiroisooxazolines, it is instructive to observe that this correlation also holds for the LMA *Ianthella* and *Aplysinella* genera. The low abundance of symbionts encumbers the description of bacterial metagenome-assembled genomes (MAGs) from LMA sponges. Consequently, the current functional relatedness of the microbiome to the holobiont metabolome is almost exclusively restricted to HMA sponges. Our data allow us to posit that the microbiomes of LMA sponges are also correlated with the holobiont metabolomes.

In conclusion, this study provides a comprehensive multi-omic description of marine sponge holobionts that harbor bromotyrosine alkaloids. We demonstrate that the microbiome architectures of these sponges are divergent, and despite the unifying presence of bromotyrosine alkaloids, the disparate microbiomes correlate with disparate metabolomes. Polar metabolomes are used to derive biosynthetic insights that include the oxidative tailoring of a nonproteinogenic amino acid, a modification that is borrowed from the biosynthetic logic for the construction of pyrrole-imidazole alkaloids. We also describe the curious conservation of betaines in sponge metabolomes, which may be primary players in mediating intra- and interorganismal interactions in benthic marine ecology. The discovery of the biogenetic routes that underlie the widespread production of bromotyrosine alkaloids will be facilitated by the development of experimental and computational tools to interrogate the genomes of eukaryotic members of the sponge holobiont, in addition to the microbial community that is traditionally accessed using contemporary sequencing and metagenome assembly workflows.

## MATERIALS AND METHODS

### DNA extraction and molecular determination of sponge phylogeny.

Metagenomic DNA was extracted from frozen sponge tissue using a protocol adapted from our previous reports (20, 29). Briefly, frozen sponge tissues were thawed overnight at 4°C, and 200 mg (wet weight) of sponge tissue was used for DNA extraction. Two regions of ribosomal DNA were amplified to be used as barcodes for genus determination. The hypervariable ITS-2 gene sequence was amplified from metagenomic DNA using GoTaq DNA polymerase with the forward primer SP58bF (5′-AATCATCGAGTCTTTGAACG-3′) and the reverse primer SP28cR (5′-CTTTTCACCTTTCCCTCA-3′). The D3-D5 region of the 28S rRNA gene was amplified using the primers NL4F (5′-GACCCGAAAGATGGTGAACTA-3′) and NL4R (5′-ACCTTGGAGACCTGATGCG-3′). A 25-μl PCR mixture was set up with 20 ng of the DNA template, 1 μl each of 10 μM forward and reverse primers, and 12.5 μl of DNA polymerase premix, with the volume adjusted to 25 μl with water. The thermocycling conditions were as follows: an initial denaturation step for 2 min at 95°C; 35 cycles each of 30 s at 95°C, 30 s at 45°C for ITS-2 and 30 s at 55°C for 28S rRNA, and 70 s at 72°C; and a final extension step for 10 min at 72°C. The PCR products were cleaned and concentrated, and clone libraries were generated using ligation-independent cloning with the pGEM-T Easy vector system kit (Promega). Three clones for each sponge amplicon were Sanger sequenced, and reads were filtered manually to remove the nucleotides derived from the vector backbone. Filtered sequences were used to search the GenBank nr/nt database using the Basic Local Alignment Search Tool (BLAST) (44). The phylogenetic tree, based on the ITS-2 sequences, was constructed in MEGA7 using default parameters for the maximum likelihood method.

### Metabolite extraction and LC/MS data collection and analyses: nonpolar metabolites.

Frozen sponge tissues were lyophilized to dryness and soaked, in technical triplicates, in a 1:1 (vol/vol) mixture of DCM-methanol (MeOH) (1 ml solvent–100 mg dry sponge tissue) for 48 h at room temperature. The organic extract was clarified by centrifugation, dried, resuspended in MeOH, and analyzed using an Agilent 1290 Infinity II ultraperformance liquid chromatography (UPLC) instrument coupled to a Bruker ImpactII ultrahigh-resolution Qq-ToF mass spectrometer equipped with an electron spray ionization (ESI) source. A Kinetex 1.7-μm C_18_ reversed-phase UPLC column (50 by 2.1 mm) was employed for chromatographic separation. Spectra were acquired in positive ionization mode at *m/z* 50 to 2,000. Active exclusion of two spectra was employed, implying that an MS^1^ ion would not be selected for MS^2^ fragmentation after two consecutive MS^2^ spectra had been recorded for it in a 0.5-min time window. For acquiring MS^2^ data, the eight most intense ions per MS^1^ spectrum were selected for fragmentation. Chromatography solvent A contained water plus 0.1% (vol/vol) formic acid, and solvent B contained MeCN plus 0.1% (vol/vol) formic acid. The flow rate was held constant at 0.5 ml/min. The elution profile employed was as follows: 5% solvent B for 3 min, a linear gradient from 5% to 50% solvent B in 5 min, 50% solvent B for 2 min, a linear gradient from 50% to 100% solvent B in 5 min, 100% solvent B for 3 min, a linear gradient from 100% to 5% solvent B in 1 min, 5% solvent B for 1 min, a linear gradient from 5% to 100% solvent B in 1 min, 100% solvent B for 3 min, a linear gradient from 100% to 5% solvent B in 1 min, and 5% solvent B for 5 min.

LC/MS data sets were converted to lock mass-corrected mzXML format using Bruker DataAnalysis software and processed using MZmine2 for feature finding (45). Data were batch processed and filtered by assigning an MS^1^ threshold for noise detection at 10,000. The following parameters were applied to extract mass spectrometric features: (i) chromatogram builder (minimum time span, 0.1 min; minimum intensity of the highest data point in the chromatogram, 1,500; *m/z* tolerance, 15 ppm), (ii) chromatogram deconvolution (local minimum search; *m/z* range for MS^2^ scan pairing, 0.025 Da; retention time range for MS^2^ scan pairing, 0.2 min), (iii) isotopic peaks grouper (*m/z* tolerance, 15 ppm; retention time tolerance, absolute, 0.1 min; maximum charge, 3; representative isotope, most intense), (iv) join aligner (*m/z* tolerance, 15 ppm; retention time tolerance, 0.1 min), (v) feature list rows filter (*m/z* range, 100 to 2,000 Da; tandem mass spectrometry [MS/MS] filter; reset peak number identification), (vi) remove duplicate filter (retention time tolerance, absolute, 0.1; *m/z* tolerance, 5 ppm), and (vii) peak finder (intensity tolerance, 0.1; retention time tolerance, absolute, 0.1; *m/z* tolerance, 15 ppm). The two output files from MZmine2 were a feature table with ion intensities (.csv file format) representing the MS^1^ feature information and a corresponding list of MS^2^ spectra linked to the MS^1^ features (.mgf file format). Principal-coordinate analysis (PCoA) was performed on the nonpolar metabolomics data. The MS^1^ feature table, obtained after processing with MZmine2 in the previous step, was used for the calculation of Bray-Curtis dissimilarity metrics using ClusterApp (q2_meatbolomics plug-in in QIIME2 [https://github.com/mwang87/q2_metabolomics]). The output files of this analysis along with the processed microbiome data were further used for performing Procrustes analysis in QIIME1, with visualization of the plots in EMPeror (46, 47).

### Isolation and structural characterization of compound 1.

Sponge dried biomass after lyophilization was soaked in DCM-MeOH (1 ml solvent–100 mg dry sponge tissue) for 48 h at room temperature. The extract was clarified by centrifugation, and 5 ml of the dried extract was loaded onto a Phenomenex Strata C_18_-E giga tube. It was then fractionated with an elution profile starting with 5% MeCN, 10% MeCN, 15% MeCN, 20% MeCN, 25% MeCN, 50% MeCN, and 100% MeCN. All the fractions were collected and dried under a vacuum and resubstituted in MeOH, and the samples were analyzed on a Bruker amaZon SL ion trap mass spectrometer to identify the extract with the molecule of interest. The desired extract was chromatographed on a Luna 5-μm C_18_ reversed-phase LC column (250 by 10 mm). Chromatography solvent A contained water plus 0.1% (vol/vol) trifluoracetic acid (TFA), and chromatography solvent B contained MeCN plus 0.1% (vol/vol) TFA. The flow rate was held constant at 2 ml/min throughout. The elution profile employed for the isolation of compound 1 was 5% solvent B for 5 min, a linear gradient from 5% to 35% solvent B in 10 min, 35% solvent B for 40 min, a linear gradient from 35% to 100% solvent B in 10 min, 100% solvent B for 3 min, a linear gradient from 100% to 5% solvent B in 1 min, 5% solvent B for 2 min, a linear gradient from 5% to 100% solvent B in 1 min, 100% solvent B for 2 min, a linear gradient from 100% to 5% solvent B in 1 min, and 5% solvent B for 3 min. Solvents were removed *in vacuo* to afford dried molecules. The molecule was dissolved in deuterated dimethyl sulfoxide (DMSO-*d*_6_) (for ^1^H NMR) and deuterated methanol (CD_3_OD) (for ^13^C NMR), followed by NMR data acquisition using an 800-MHz Bruker Avance III HD instrument.

### Metabolite extraction and LC/MS data collection and analyses: polar metabolites.

In a 2-ml Eppendorf safe-lock tube, lyophilized sponge tissues were homogenized with two tungsten carbide beads in a Qiagen TissueLyser II instrument at 20 Hz for 20 min, in 2 cycles of 10 min each. Samples were extracted with 80% MeOH (4 ml of solvent per 100 mg of sample), sonicated for 30 min in an ice bath, and centrifuged at 16,000 × *g* for 30 min. The supernatant was transferred to an autosampler vial for further analysis. For quality control, a pooled sample was created by mixing an equal volume from each sample extract. A sample blank was created according to the above-described procedure with no sample.

LC/MS data were acquired using a Waters Corporation Acquity UPLC BEH amide column (2.1 by 150 mm, 1.7-μm particle size) coupled to a high-resolution accurate-mass Orbitrap ID-X tribrid mass spectrometer. The chromatographic method for sample analysis involved elution with a 20:80 mixture of water-MeCN with 10 mM ammonium formate and 0.1% formic acid (mobile phase A) and MeCN with 0.1% formic acid (mobile phase B) using the following gradient program: 0 min 5% mobile phase A, 0.5 min 5% mobile phase A, 8 min 60% mobile phase A, 10.4 min 60% mobile phase A, 10.5 min 5% mobile phase A, and 14 min 5% mobile phase A. The flow rate was set at 0.4 ml/min. The column temperature was set to 40°C, and the injection volume was 0.5 μl.

The Orbitrap ID-X instrument is a tribrid spectrometer that utilizes quadrupole isolation with dual detectors, an orbitrap, and an ion trap, with a maximum resolving power of 500,000 full width at half-maximum (FWHM) at *m/z* 200 and a mass accuracy of <1 ppm with the use of an internal calibrant. The ESI source was operated at a vaporizer temperature of 275°C, a spray voltage of 3.5 kV, and sheath, auxiliary, and sweep gas flows of 40, 8, and 1 (arbitrary units), respectively. UPLC-MS^2^ experiments were performed by acquiring mass spectra with data-dependent acquisition (DDA) and targeted MS/MS (tMS^2^) acquisition. DDA methods collected full-scan data with a resolution of 120,000, and the dd-MS^2^ spectra were collected at a resolution of 30,000 with an isolation window of 0.8 *m/z* and a cycle time of 0.6 s. Dynamic exclusion was set to exclude MS/MS acquisition of a precursor if the precursor was acquired twice within a 3-s window. The exclusion duration was 4 s. Mass tolerance was set at 3 ppm. dd-MS^2^ ions were activated by high-energy collisional dissociation (HCD) at normalized collision energies of 40% and a collision-induced dissociation (CID) collision energy of 35%, and product ions were measured in the orbitrap at a resolution of 30,000. tMS^2^ methods acquired full-scan data at a 30,000 resolution and activated product ions from the inclusion list with an isolation window of 0.8 *m/z* and HCD activation at 30% energy ± 60%. The inclusion list loop control was set to *n* = 4.

The raw data files were converted to mzXML format using ProteoWizard msConvert. These mzXML files were batch processed using MZmine2 for feature finding as described above for the nonpolar metabolome. The MS^1^ data were filtered by assigning a threshold level for noise detection at 10,000; no threshold was used for filtering MS^2^ spectra. The feature list rows filter was set to an *m/z* range of 70 to 2,000. All the other parameters were the same as those used for nonpolar data processing. PCoA and Procrustes analyses were performed using the MS^1^ feature table as described above for nonpolar data. In addition, the DDA and tMS^2^ raw data files were manually curated to obtain extracted ion chromatograms (EICs) and MS^2^ fragmentation spectra, respectively. Retention times and MS^2^ spectra were accessed for the desired molecules in the sponge extract samples and compared to those of the corresponding synthetic standards. The relative abundances of the molecules of study were manually derived from the area under the EICs (plotted within a 2-ppm error). Histograms were constructed from these areas using Origin 2020 software. A volcano plot was generated from the polar feature table output of MZmine2 using Metaboanalyst.

### Synthesis and characterization of standards.

Molecules 2 to 5 were synthesized according to literature procedures (48–51).

For (*S*)-2-amino-3-(3-bromo-4-hydroxyphenyl)-propanoic acid (compound 2), to a stirred solution of l-tyrosine (500 mg; 2.76 mmol) in 25 ml MeOH at 0°C under argon, SOCl_2_ (1,000 μl; 13.79 mmol; 5 eq) was added slowly, and the reaction mixture was maintained at the same temperature for 15 min. The reaction mixture was then allowed to stir overnight at room temperature under argon. The reaction mixture was then concentrated under a vacuum and washed with 20 ml MeOH three times, and the crude was dried under a vacuum to obtain l-tyrosine methyl ester. Monobromination of this crude methyl ester (100 mg; 0.5 mmol; 1 eq) was achieved with *N*-bromosuccinimide (NBS) (118 mg; 0.65 mmol; 1.29 eq) in 10 ml MeOH with stirring at room temperature for 24 h. The solvent was removed under a vacuum, and the crude reaction mixture was partitioned with ethyl acetate (EtOAc) and H_2_O. The aqueous phase was recovered and dried, the crude material was dissolved in 5 ml MeOH-H_2_O-tetrahydrofuran (THF) (1:1:1), and K_2_CO_3_ (100 mg; 1.69 mmol; 3 eq) was added. The reaction mixture was stirred for 24 h at room temperature. The pH was adjusted to 6, and conversion was monitored by thin-layer chromatography (TLC) (mobile phase, nBuOH-acetic acid [AcOH]-H_2_O [4:1:1]). Compound 2 was obtained after high-performance liquid chromatography (HPLC) purification. ^1^H NMR (see Fig. S4 in the supplemental material) (800 MHz, methanol-*d*_4_), δ 7.43 (d, *J *= 2.1 Hz, 1H), 7.10 (dd, *J *= 8.3, 2.2 Hz, 1H), 6.88 (d, *J *= 8.3 Hz, 1H), 3.95 (ddd, *J *= 8.2, 4.9, 1.0 Hz, 1H), 3.21 to 3.18 (m, 1H), 3.00 to 2.97 (m, 1H).

For (*S*)-2-amino-3-(3-bromo-4-methoxyphenyl)-propanoic acid (compound 3), to a stirred solution of *O*-methyl-l-tyrosine (300 mg; 1.54 mmol; 1 eq) in 25 ml of cooled MeOH, SOCl_2_ (550 μl; 7.68 mmol; 5 eq) was added slowly, and the reaction mixture was maintained at 0°C for 15 min. The remainder of the procedure was the same as the one described above for synthesizing compound 3. ^1^H NMR (Fig. S5) (800 MHz, methanol-*d*_4_), δ 7.50 (d, *J *= 2.1 Hz, 1H), 7.24 (dd, *J *= 8.4, 2.2 Hz, 1H), 7.02 (d, *J *= 8.4 Hz, 1H), 4.12 (dd, *J *= 7.7, 5.4 Hz, 1H), 3.87 (s, 3H), 3.23 (dd, *J *= 14.7, 5.4 Hz, 1H), 3.07 (dd, *J *= 14.7, 7.7 Hz, 1H).

(*S*)-2-amino-3-(3,5-dibromo-4-hydroxyphenyl)-propanoic acid (compound 4) was obtained in the same way as described above for compound 2 using NBS (187 mg; 1.05 mmol; 2.2 eq). ^1^H NMR (Fig. S6) (800 MHz, methanol-*d*_4_), δ 7.45 (s, 2H), 4.15 (dd, *J *= 7.7, 5.4 Hz, 1H), 3.19 (dd, *J *= 14.8, 5.4 Hz, 1H), 3.04 (dd, *J *= 14.8, 7.6 Hz, 1H).

For (*S*)-2-amino-3-(3,5-dibromo-4-methoxyphenyl)-propanoic acid (compound 5), the crude reaction mixture of *O*-methyl-l-tyrosine methyl ester (200 mg; 0.73 mmol; 1 eq) was dissolved in 10 ml MeOH, followed by the addition of Boc_2_O (185 mg; 0.85 mmol; 1.16 eq) and TEA (400 μl). The reaction mixture was stirred for 24 h at room temperature. The solvent was evaporated under a vacuum, and the pH was adjusted to 3. This crude extract was taken forward for the *o*-methylation reaction. To a stirred solution of crude compound 5a (Fig. S7) (200 mg; 0.44 mmol; 1eq) in 10 ml acetone, K_2_CO_3_ (106 mg; 0.77 mmol; 1.74 eq) and MeI (100 μl; 1.6 mmol; 3.62 eq) were added in the respective order. The mixture was stirred for 18 h at room temperature. The solvent was removed, and the reaction mixture was purified via column chromatography using hexane-EtOAc (3:1). The pure material 5b was dissolved in 5 ml DCM at 0°C and stirred for 10 min, followed by the addition of 1 ml TFA with stirring, and the reaction was allowed to proceed for 24 h at room temperature. The product obtained in the previous step, compound 5c, was dissolved in a 5-ml mixture of MeOH-H_2_O-THF (1:1:1), and K_2_CO_3_ (113 mg; 0.82 mmol; 3 eq) was added. The reaction mixture was stirred for 24 h at room temperature. The pH-adjusted crude extract was then purified by reversed-phase HPLC (RP-HPLC) using a mobile phase mixture of MeCN (0.1% TFA) and H_2_O (0.1% TFA) to obtain pure compound 5. ^1^H NMR (Fig. S7) (800 MHz, methanol-*d*_4_), δ 7.55 (s, 2H), 4.27 to 4.26 (m, 1H), 3.86 (s, 3H), 3.25 (dd, *J *= 14.7, 5.8 Hz, 1H), 3.12 (dd, *J *= 14.7, 7.5 Hz, 1H).

### Microbiome sequencing and analyses.

The procedure for querying the sponge-associated microbiome was based on our previous reports (20, 29). Next-generation sequencing of the v4 region of the 16S rRNA gene on the Illumina MiSeq platform was used. Briefly, the region of interest was amplified by the primer pair 515F/806R, which are barcoded and appended with Illumina-specific adaptors. PCR mixtures contained 1 μl 20 ng/μl template DNA, 0.5 μl each 20 μM forward and reverse primer, 0.5 μl 10 nM deoxynucleoside triphosphates (dNTPs), 0.25 μl Q5 high-fidelity DNA polymerase, reaction buffer, and molecular-biology-grade water to a final volume of 25 μl. The thermocycling conditions were as follows: an initial denaturation step for 30 s at 98°C; 35 cycles each of 30 s at 98°C, 30 s at 50°C, and 20 s at 72°C; and a final extension step for 2 min at 72°C. Purified and concentrated PCR amplicons were pooled in equimolar concentrations for sequencing on the Illumina MiSeq platform. The raw sequence reads were demultiplexed and sequence variants (SVs) were generated by QIIME2 using the qiime tools import script, the qiime demux script, and the DADA2 plug-in, respectively (52, 53). Based on quality scores, the forward and reverse reads were truncated at 150 bp using the qiime dada2 denoise script. Taxonomy was assigned using the SILVA pretrained classifier using the qiime feature classifier plug-in (54). The qiime taxa barplot script was used to generate the bar plots representing the taxonomic distribution.

The α-diversity of the microbiome was quantified by computing Shannon index values using the q2_diversity plug-in in QIIME2. To highlight the divergence of the microbiome on moving from level 2 “phylum” classification to level 6 “genus” among the different sponge genera within the study, a microbiome network was generated based on the SV feature table generated by QIIME2. The vegan library of the R package was used to generate the dissimilarity matrix and the corresponding network. The vegdist() function was used to calculate the Bray-Curtis distances between the sponge samples. The distances were converted to a matrix using the as.matrix() function. In order to build a network, the dissimilarity matrix was converted to an adjacency matrix using the graph.adjacency() function. A threshold of 0.6 was used for the conversion to the adjacency matrix, implying that SVs with a dissimilarity index of ≤0.6 will be connected to each other. The network thus generated was exported from RStudio in graphml format and visualized in Cytoscape. Three networks were generated at the phylum (level 2), order (level 4), and genus (level 6) levels. Procrustes analysis was performed on both the metabolomics (as described above) and microbiome data sets using QIIME1. For the microbiome data, the unweighted UniFrac metric was used to generate the pairwise dissimilarity matrix to be used in Procrustes analysis (55).

Sponge specimens from the Solomon Islands were acquired via export permit rp/2017/003.

### Data availability.

Sponge 28S rRNA sequences and ITS-2 sequences were submitted to GenBank under the accession numbers MW377651 to MW377670 and MW377671 to MW377687, respectively. The 16S amplicon sequencing data are available under BioProject accession number PRJNA686181. LC/MS data were deposited in the UCSD Center for Computational Mass Spectrometry database with the MassIVE identifier MSV000086608.
