# Supplementary material for: Presence of Bromotyrosine Alkaloids in Marine Sponges Is Independent of Metabolomic and Microbiome Architectures
Source: mSystems. 2021 Mar 16;6(2):e01387-20. doi: 10.1128/mSystems.01387-20 (PMC8547014; doi:10.1128/mSystems.01387-20)
Supplement: TABLE S1 [file msystems.01387-20-st001.docx]

| **Order** | **Family** | **Genera** | **Natural product class** | **Location** |
| --- | --- | --- | --- | --- |
| Verongiida | Aplysinidae | *Aplysina* | Spiroisooxazolines^2^ | Florida Keys |
| Verongiida | Aplysinidae | *Aplysina* | Spiroisooxazolines^2^ | Florida Keys |
| Verongiida | Aplysinidae | *Verongula* | Spiroisooxazolines^2^ | Florida Keys |
| Verongiida | Aplysinidae | *Aiolochroia* | Spiroisooxazolines^2^ | Florida Keys |
| Verongiida | Aplysinidae | *Aplysina* | Spiroisooxazolines^2^ | Florida Keys |
| Verongiida | Aplysinidae | *Aplysina*^1^ | Spiroisooxazolines^2^ | Florida Keys |
| Verongiida | Aplysinidae | *Verongula*^1^ | Spiroisooxazolines^2^ | Florida Keys |
| Verongiida | Aplysinidae | *Verongula*^1^ | Spiroisooxazolines^2^ | Florida Keys |
| Verongiida | Aplysinidae | *Aplysina* | Spiroisooxazolines^2^ | Puerto Rico |
| Verongiida | Aplysinidae | *Aiolochroia* | Spiroisooxazolines^2^ | Puerto Rico |
| Verongiida | Pseudoceratinidae | *Pseudoceratina* | Spiroisooxazolines^2^ | Puerto Rico |
| Verongiida | Pseudoceratinidae | *Pseudoceratina*^1^ | Spiroisooxazolines^2^ | Puerto Rico |
| Verongiida | Ianthellidae | *Ianthella* | Bastadins^3^ | Guam |
| Verongiida | Ianthellidae | *Aplysinella* | Psammaplins^4^ | Guam |
| Poecilosclerida | Iotrochotidae | *Iotrochota* | Spiroisooxazolines^2^ | Solomon Islands |
| Dictyoceratida | Thorectidae | *Hyrtios* | Spiroisooxazolines^2^ | Solomon Islands |
| Verongiida | Pseudoceratinidae | *Pseudoceratina*^1^ | Spiroisooxazolines^2^ | Solomon Islands |
| Verongiida | Aplysinellidae | *Suberea* | Spiroisooxazolines^2^ | Solomon Islands |
| Poecilosclerida | Iotrochotidae | *Iotrochota*^1^ | Spiroisooxazolines^2^ | Solomon Islands |
| Verongiida | Aplysinellidae | *Suberea* | Spiroisooxazolines^2^ | Solomon Islands |
| Verongiida | Ianthellidae | *Ianthella* | Bastadins^3^ | Solomon Islands |
| Verongiida | Aplysinellidae | *Aplysinella* | Psammaplins^4^ | Solomon Islands |
| **Control sponges** | | | | |
| Dictyoceratida | Thorectidae | *Smenospongia* | Brominated indoles | Florida Keys |
| Scopalinida | Scopalinidae | *Stylissa* | Pyrrole-imidazole alkaloids^5^ | Guam |
| Agelasida | Agelasidae | *Agelas* | Pyrrole-imidazole alkaloids^5^ | Solomon Islands |
| Dictyoceratida | Dysideidae | *Dysidea* | Barbaleucamides | Solomon Islands |

^1^ITS-2 amplicons for these specimens could not be obtained, sponge phylogenetic assignment is based on 28S rRNA sequence homology

^2^Natural product purealidin L was detected in these sponge specimens

^3^Natural product bastadins 1 was detected in these sponge specimens

^4^Natural product psammaplin A was detected in these sponge specimens

^5^Natural product oroidin was detected in these sponge specimens
