# Supplementary figures and images for: Presence of Bromotyrosine Alkaloids in Marine Sponges Is Independent of Metabolomic and Microbiome Architectures
Source: mSystems. 2021 Mar 16;6(2):e01387-20. doi: 10.1128/mSystems.01387-20 (PMC8547014; doi:10.1128/mSystems.01387-20)

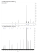

Supplement: FIG S3 [file msystems.01387-20-sf003.pdf]
